# Identification of vaginal microbiome associated with IVF pregnancy

**DOI:** 10.1038/s41598-022-10933-2

**Published:** 2022-04-26

**Authors:** B. Lledo, A. Fuentes, F. M. Lozano, A. Cascales, R. Morales, M. Hortal, F. Sellers, A. Palacios-Marques, R. Bermejo, F. Quereda, J. C. Martínez-Escoriza, R. Bernabeu, A. Bernabeu

**Affiliations:** 1 Molecular Biology, Instituto Bernabeu of Fertility and Gynecology, Avda. Albufereta, 31, 03016 Alicante, Spain; 2 Reproductive Medicine, Instituto Bernabeu of Fertility and Gynecology, 03016 Alicante, Spain; 3 Obstetrics, Instituto Bernabeu of Fertility and Gynecology, 03016 Alicante, Spain; 4 Division of Gynecology, Hospital Universitario San Juan de Alicante, 03550 Alicante, Spain; 5 Division of Gynecology, School of Medicine, Miguel Hernández University, Alicante, Spain; 6 Obstetrics and Gynecology, Hospital General Universitario de Alicante, 03010 Alicante, Spain; 7 ISABIAL (Instituto de Investigación Sanitaria y Biomédica de Alicante), Alicante, Spain

**Keywords:** Microbial genetics, Infertility

## Abstract

The factors that cause a preterm birth (PTB) are not completely understood up to date. Moreover, PTB is more common in pregnancies achieved by in-vitro fertilization (IVF) than in spontaneous pregnancies. Our aim was to compare the composition of vaginal microbiome at 12 weeks of gestation between women who conceived naturally or through IVF in order to study whether IVF PTB-risk could be related to vaginal microbiome composition. We performed an observational, prospective and multicentre study among two public hospitals and a fertility private clinic in Spain. Vaginal swabs from 64 pregnant women at 12 weeks of gestation were collected to analyse the microbiome composition by sequencing the V3–V4 region of the 16S rRNA. Our results showed that the vaginal microbiome signature at 12 weeks of pregnancy was different from women who conceived naturally or through IVF. The beta diversity and the genus composition were different between both cohorts. *Gardnerella*, *Neisseria*, *Prevotella*, and *Staphylococcus* genus were enriched genus in the vaginal microbiome from the IVF group, allowing us to create a balance model to predict both cohorts. Moreover, at species level the *L. iners* abundance was higher and *L. gasseri* was lower in the IVF group. As a conclusion, our findings were consistent with a proposed framework in which IVF pregnancy are related to risk for preterm birth (PTB) suggesting vaginal microbiome could be the reason to the relation between IVF pregnancy and risk for PTB.

## Introduction

The human microbiota plays a key role in promoting individual’s health. In fact, a microbial dysbiosis can result in many pathological conditions. In healthy women, the vaginal microbiome is dominated by a homogeneous variety of *Lactobacillus* species (*L. iners*, *L. crispatus*, *L. gasseri* and *L. jensenii*). *Lactobacillus* species produce lactic acid in the vagina that helps preserving vaginal acidic pH, which acts as a bactericidal against pathogenic bacteria. In contrast, a vaginal microbiome with high diversity of species, as observed in bacterial vaginosis, involves a risk for infections and pelvic inflammatory disease. The vaginal microbiome composition can vary with age, reproductive cycling and race^1^. Furthermore, the vaginal microbiome of pregnant women differs from that of women in other life stages. Studies have shown that the microbiome composition is associated not only with reproductive problems but also with obstetric and perinatal outcomes^2^.

It has been observed that fertility factors associated with levels of reproductive hormones modify the vaginal microbiome in reproductive-aged women, showing fewer *Lactobacillus* species in women with higher E2 (estradiol) and LH (Luteinizing hormone) and higher concentration of pathogenic bacteria^3^. Thus, evidence suggests that the microbiome could influence infertility, as those dominated by *Lactobacillus* species are associated with the achievement of pregnancy^4–6^. In addition, vaginal dysbiosis is significantly associated with higher early pregnancy loss rate among women undergoing IVF and a significant negative impact on clinical pregnancy rates per embryo transfer^7^.

On the other hand, some reports have suggested that the composition of the vaginal microbiome has impact on obstetric complications such as preterm birth (PTB). Women who delivered preterm have significantly lower vaginal levels of *Lactobacillus crispatus* and higher abundance of *Prevotella* species^8^. Haque et al., concluded that there are significant differences between “first-trimester” vaginal microbiomes obtained from women with term and preterm outcomes^9^.

Until now, the factors involved in preterm birth (PTB) are not completely understood. The factors typically associated with increased PTB risk are: genetic predisposition, maternal risk factors (e.g., age, alcohol intake, smoking, reproductive history), intrauterine infections, urinary tract infections, etc. Moreover, in pregnancies conceived by in-vitro fertilization (IVF) PTB is more common as compared with those conceived naturally. A recent meta-analysis concludes that the risk of PTB in singleton pregnancies resulting from IVF/ICSI is significantly greater than that in spontaneously conceived singletons^10^.

The primary aim of our study was to compare the vaginal microbiome composition at 12 weeks of gestation, between women who conceived naturally or through IVF, in order to state if IVF PTB risk could be related to vaginal microbiome composition.

## Results

### Population

Sixty-four women were enrolled in the study. Socio-demographic characteristics of women who had spontaneous pregnancies (n = 30) and women who had IVF pregnancies (n = 34) are summarized in Table 1. There were no significant differences in body mass index (BMI), previous pregnancies, previous gynaecological pathologies, previous uterine surgery (C-section not included) and previous bad obstetrical history (miscarriages, ectopic pregnancy, pre-eclampsia or fetal death) (p > 0.05). Average maternal age, previous healthy babies born and previous infertility history were significantly different between groups. Average maternal age was 33.8 ± 4.57 year (minimum: 19 year, maximum: 44 year) and 38.9 ± 4.26 year (minimum: 30 year, maximum: 46 year) for women in the spontaneous pregnancies and IVF cohorts, respectively (p = 0.0001). The percentage of patients from the IVF group who had previous history of infertility was 82.35 vs 6.67 from the spontaneous group (p = 0.0001). Also, 53.33% of patients belonging to the spontaneous cohort had a previous healthy baby born vs 17.65% from the IVF cohort (p = 0.0027). These three factors are related and then considered as covariables for further statistical analysis because they are directly related to the definition of the group of patients. Patients from the IVF group were older, with previous history of infertility and fewer healthy babies born because they needed to undergo an IVF treatment. Finally, according to the current pregnancy, no significant differences were reported in pregnancy complications (hypertension, preeclampsia, gestational diabetes, growth retardation, chorioamnionitis and metrorragy) amniotic sac rupture, preterm birth (babies born alive before 37 weeks of pregnancy are completed), healthy babies born (pregnancy that results in a baby weighing at least 2.5 kg who has no birth defects) and neonatal complications (infections, neonatal respiratory distress and intensive care unit) (p > 0.05).

**Table 1:** Patient demographic and clinical information.

	All women n = 64	Spontaneous pregnancy n = 30	IVF pregnancy n = 34	p
Maternal age (year) (average ± SD)	36.2 ± 4.95	33.8 ± 4.57	38.9 ± 4.26	**0.0001**
Body mass index (BMI) (average ± SD)	24.28 ± 5.99	24.87 ± 5.85	23.73 ± 4.07	0.3776
Previous pregnancies (%)	56.25	56.67	55.88	0.9497
No previous gynaecological pathologies (%)	84.4	90.0	79.41	0.430
Previous uterine surgery (%)	6.25	6.67	5.88	0.8971
Previous c-section (%)	22.72	29.41	20.0	0.572
Previous bad obstetrical history (%)	37.5	23.30	43.33	0.1572
Previous healthy babies (%)	34.38	53.33	17.65	**0.0027**
Previous infertility history (%)	46.88	6.67	82.35	**0.0001**
Pregnancy complications (%)	38.10	40.0	36.4	0.375
Amniotic sac rupture (%)	35.48	31.03	39.40	0.4924
Preterm birth (%)	12.50	6.67 (2)	17.64 (6)	0.2031
Healthy baby born (%)	98.4	100	96.97	0.5221
Neonatal complications (%)	8.20	7.14	9.38	0.6187

p-value < 0.05 are given in bold.

### IVF or spontaneous pregnancy and microbial diversity

**Figure 1:**
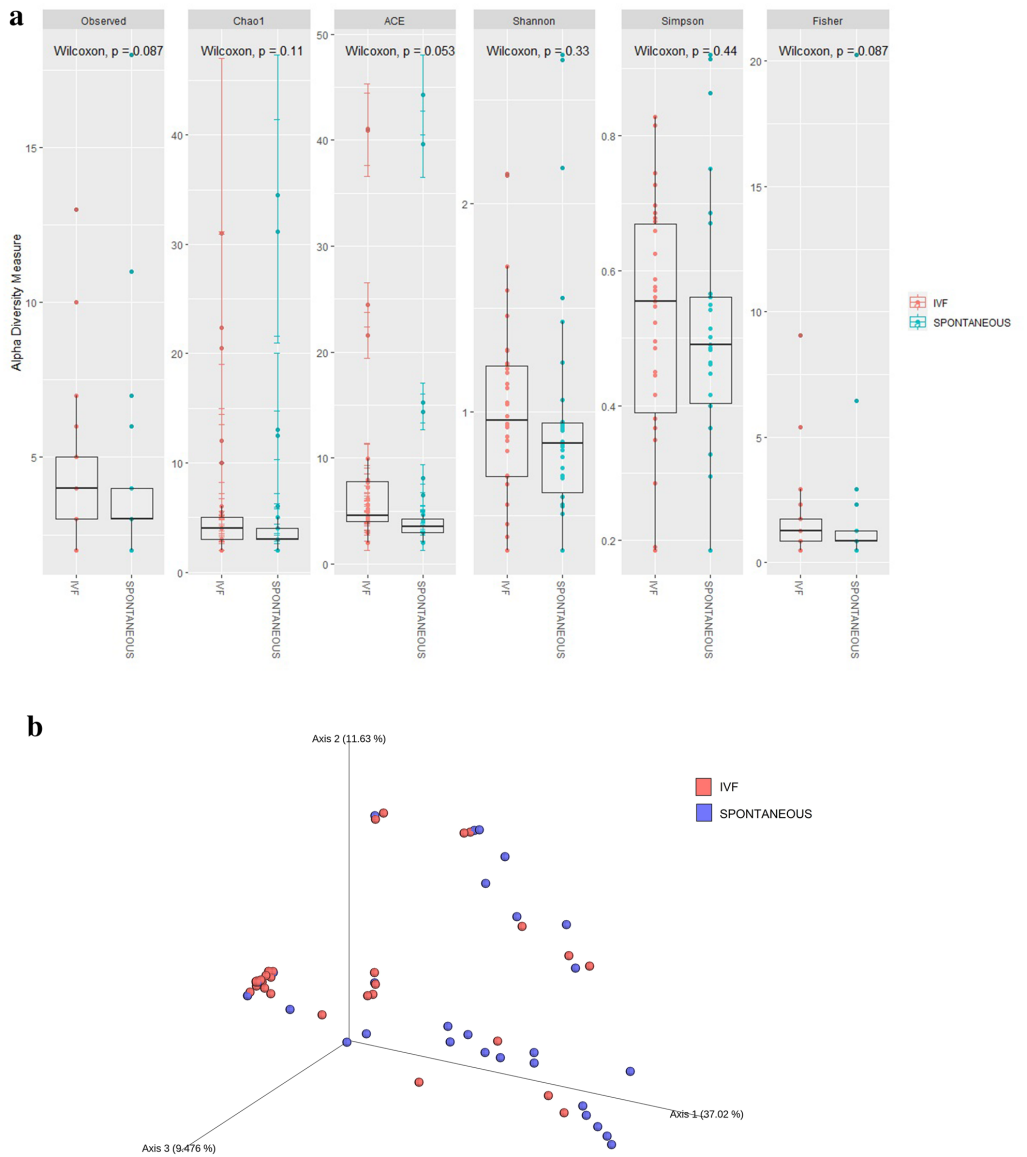
Diversity analysis. (**a**) Alpha diversity indexes boxplots between both cohorts (IVF vs spontaneous). Different indexes are shown. Using two-sided Wilcoxon’s test followed by a P value adjustment using Bonferroni’s correction with 5% FDR. Boxes show median and interquartile range. (**b**) Beta diversity. Principal coordinates analysis (PCOA) between IVF and spontaneous cohorts. The figure shows the three-dimensional diagram of PCOA, in which each dot represents a sample, and each colour represents a group: red for spontaneous pregnancy group and blue for IVF group. PC1 is the principal coordinate component causing the largest difference in samples, with an explanatory value of 37.02%. PC2 and PC3 were next, with an explanatory value of 11.63% and 9.47%, respectively.

Alpha diversity at genus level was applied for analysing complexity of taxa diversity for a sample through several indexes (Fig. 1a). Although a trend toward an increased microbial diversity was evident in the IVF pregnancies compared to the spontaneous pregnancies, no statistically significant differences in richness were observed using either Observed, Chao1, Shannon, Simpson and Fisher indexes. As for ACE (Abundance-based Coverage Estimator) index, the p value obtained was near to be statistically significant, p = 0.053. ACE index was used to estimate richness (measurement of OTUs/genus expected in samples given all the bacterial that were identified in the samples)^11^, the greater the ACE index, the higher the expected species richness of the microbiota. Moreover, ACE mainly depends on the number of rare OTUs/genus. According to our result vaginal microbiome at 12 weeks of gestation in patients from the IVF group had higher ACE index than patients from spontaneous pregnancy group (p = 0.053). Given that IVF and spontaneous pregnancy group were different with respect to maternal age, alpha diversity was evaluated for age. No significant differences were reported for Shannon (p = 0.450) and Chao1 (p = 0.582) indexes and maternal age (Fig. S1).

Following alpha analysis and in order to further display differences in the diversity of species among samples, principal coordinate analysis (PCOA) based on phylogenetic unweighted unifrac distances at genus level was used to display differences among samples. If the two samples are close together, the species composition of the two samples is similar. A significant separation in bacterial community composition between IVF and spontaneous cohorts was revealed according to the Fig. 1b. When we compared the beta diversity of vaginal microbial at 12 weeks of gestation by cohort using PERMANOVA a significant difference was obtained (p = 0.001).

### Vaginal microbiome profile of spontaneous vs IVF pregnant women and prediction model at genus level

**Figure 2:**
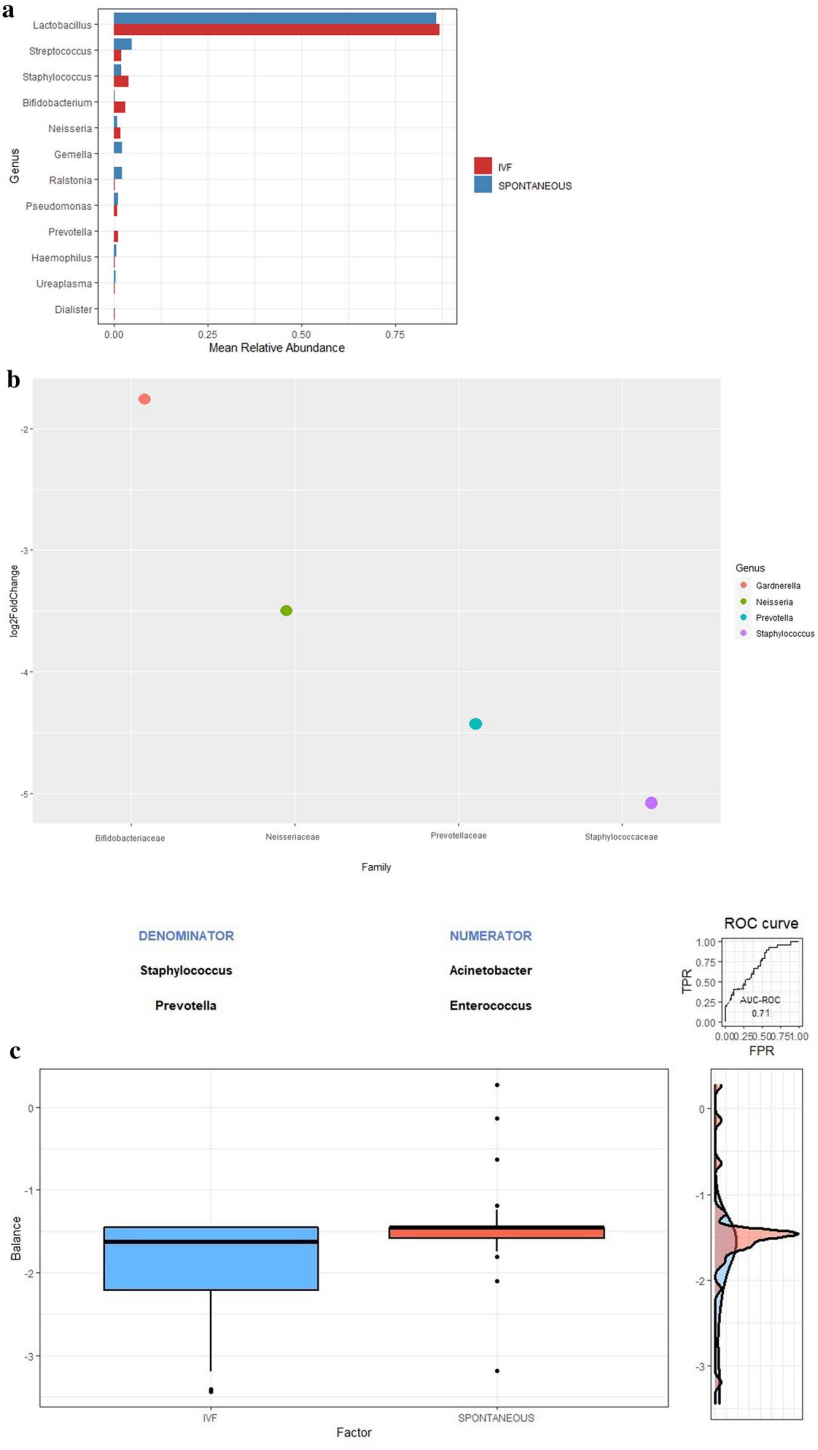
Composition of the bacterial community at the genus level. (**a**) By cohort. (**b**) By differential taxa abundances. The plot shows the log2-fold changes for genus-level bacterial that were statistically significant at the 0.01 level using the Wald test with Benjamini–Hochberg adjustment as implemented in DESeq2. Each data point represents a genus-level (y-axis) identified as significantly different along with the log2 fold change (x-axis). Negative values indicate the log2 fold change for taxa underrepresented in vaginal microbiome at 12 weeks from patients with a spontaneous pregnancy. (**c**) By global balance. The two groups of taxa that form the global balance are specified at the top of the plot. The box plot represents the distribution of the balance scores for IVF and spontaneous pregnancies. The right part of the figure contains the ROC curve with its AUC value (0.71) and the density curve for each group.

The taxonomic microbial profiling of the vaginal swabs of pregnant women from the spontaneous group at 12 weeks of gestation, showed a community represented by 10 genera that accounted for more than 99% of the total sequence reads. Among them, the *Lactobacillus* genus (85.58%) was followed by *Streptococcus* (4.57%), *Gemella* (2.17%), *Ralstonia* (1.6%) and *Staphylococcus* (1.5%) (Fig. 2a). For pregnant women from the IVF cohort *Lactobacillus* genus (86.93%) was followed by *Staphylococcus* (3.57%), *Bifidobacterium* (3.02%), *Prevotella* (1.6%) and *Neisseria* (1.5%) (Fig. 2a).

To investigate whether there was an association between individual taxa and spontaneous or IVF pregnancies, the abundance of each genus was evaluated. Empirical Bayes techniques were employed to analyse differences in the abundance between two groups, estimating priors for log fold change and dispersion, and to calculate posterior estimates for these quantities. We screened out the genus that caused the difference in the composition of the two cohorts of samples (Fig. 2b). *Gardnerella, Neisseria*, *Prevotella*, and *Staphylococcus* were significantly enriched in the vaginal microbiome from IVF pregnant women at 12 weeks of gestation. Regarding the *Lactobacillus* abundance, no difference was reported as both group included pregnant women in which *Lactobacillus* was the most prevalent genus.

The two groups of genus defining the global balance, or microbial signature, for IVF pregnancies were X +  = {*Acinetobacter*, *Enterococcus*} and X−  = {*Staphylococcus*, *Prevotella*}. Figure 2c presents the distribution of the microbial signature values for IVF and spontaneous pregnancies. Women with IVF pregnancies had lower balance scores than the spontaneous pregnancy group, which means that there are lower relative abundances of taxa in group X+ than in group X−. These results agree with our previous genus difference composition between groups, as *Staphylococcus* and *Prevotella* have also been previously identified as more abundant in IVF pregnancies than in natural conceptions. The discrimination value of the identified balance is important, with an apparent AUC value of 0.71 with 1 repeat of fivefold cross-validation.

### *Lactobacillus* species profile and community state type (CST) distribution of spontaneous vs IVF pregnant women

**Figure 3:**
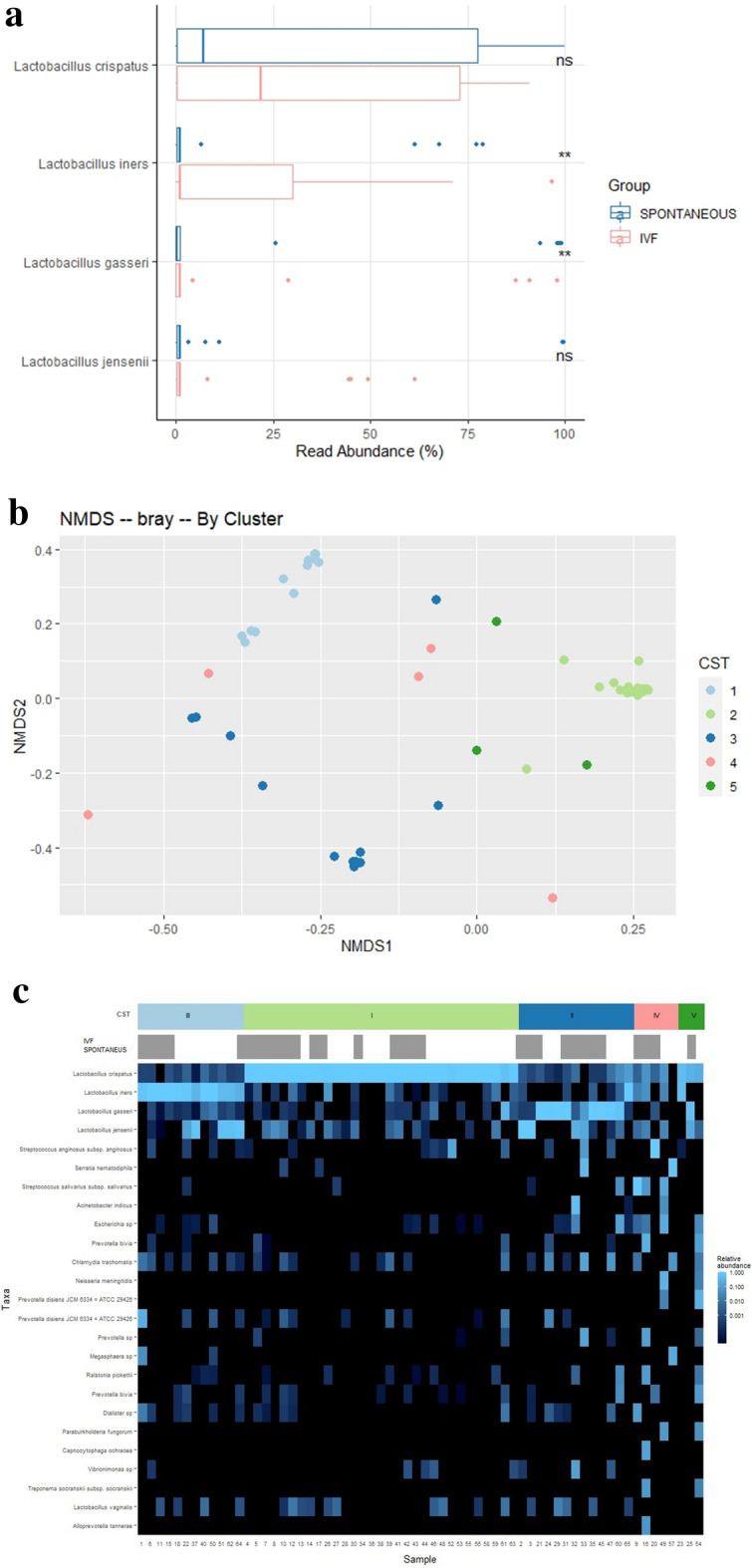
Composition of the bacterial community. (**a**) At the *Lactobacillus* level. Boxplots showing the % of read abundance of *Lactobacillus* genus in vaginal microbiome at 12 weeks in the IVF vs in the spontaneous pregnancy group. Asterisks show statistical significant two-sided Wilcoxon’s test. (**b**) At the CST level. Principal Coordinate Analysis (PCoA) ordination based on Bray Curtis non-metric multidimensional scaling (NMDS) dissimilarities permutational multivariate analysis of variance (PERMANOVA) 999 permutations; p-value < 0.001). Samples belonging to different CSTs are indicated with different colour dots. (**c**) A heatmap of relative abundance of the top 25 species found in the vaginal samples of pregnant women at 12 weeks included in our study. Each vertical line represents 1 sample. *CST*, community state type, *IVF* and spontaneous group.

At the species taxonomic level, we evaluated the association between the relative abundance of *Lactobacillus* species and pregnant women from the spontaneous pregnancies and the IVF group. We analysed the top 4 more abundant *Lactobacillus* species present in our samples. *Lactobacillus iners* showed a higher abundance in vaginal microbiome at 12 weeks of gestation in women from the IVF group (15.2%) vs spontaneous (9.8%) pregnancy group (p = 0.002). On the other hand, *Lactobacillus gasseri* showed a lower abundance in vaginal microbiome at 12 weeks from women belonged to IVF (9.2%) vs the spontaneous (13.8%) pregnancy group (p = 0.005) (Fig. 3a).

To deeper understand vaginal microbiota composition at 12 weeks of pregnancy and to define microbial profiles associated with spontaneous or IVF pregnancies, we tried to identify different samples cluster at species level. Principal Coordinate Analysis (PCoA) ordination based on Bray Curtis dissimilarities was used for cluster finding (Fig. 3b). We identified five cluster that correlated with the five major vaginal community state types according to the CST. Figure 3c shows a heatmap of the top 25 species found in the vaginal samples of pregnant women at 12 weeks included in our study grouped by CST.

Overall, the distribution of vaginal CST at 12 weeks of pregnancy among the 64 participants in our study (Table S1) was: nearly half of the women had a vaginal microbiome classified as *L. crispatus* dominated microbiota (CST I), approximately 20% had CST III (*L. iners* dominated) and other 20% had CST II (*L. gasseri* dominated). *Lactobacillus*-depleted microbiota, defined as CST-IV was found in 7.8% of pregnant women at 12 weeks. No significance differences in CST distribution were reported in women, regardless if belonged to spontaneous or IVF pregnancies. According to the species level compositional previous result, CST I, II, and V were combined into a single category creating three CST categories: non-iners *Lactobacillus* dominated (CST I, II, V), *Lactobacillus iners* dominated (CST III), non-*Lactobacillus* dominated or Diverse (CST IV) (Fig. S2). Sixty-seven (8/12) percent of patients which vaginal microbiome at 12 weeks was *Lactobacillus iners* dominated belonged to the IVF pregnancy group.

## Discussion

To our knowledge this is the first study that compares the vaginal microbiome at 12 weeks of pregnant women who achieved a pregnancy spontaneously or using IVF techniques. The main finding of this study showed that vaginal microbiome signature at 12 weeks of pregnancy is different between both groups of patients. The beta diversity and the genus composition are different between both cohorts. It allowed us to create a balance model to predict the group that they belong to. Moreover, at species level the *L. iners* abundance was higher and *L. gasseri* lower in the IVF group. Furthermore, the IVF cohort showed a signature of PTB including several taxa that have previously been implicated in adverse outcomes of pregnancy, including premature delivery^12^. Thus, our findings are consistent with a proposed framework in which IVF pregnancies is related to risk for PTB suggesting vaginal microbiome could be the reason to link IVF pregnancies to risk for PTB.

The present study compared the vaginal microbiome of 64 pregnant women. The cohorts were comparable in terms of clinical characteristics. However, differences in maternal age, number of previous healthy babies and previous history of infertility were observed. This is not surprising because the patients that were included in the IVF group were requiring assisted reproduction techniques to have a child. The vaginal microbiome of both cohorts was dominated by *Lactobacillus,* as reported by previous studies associated with pregnancy^13^. Indeed, lactobacilli are key players in the vagina, protecting women at a reproductive age against the colonization of potential exogenous pathogens, probably through the production of lactic acid and the decrease of pH^14^. As for, preterm birth no significances differences between groups were reported although there was a higher percentage of preterm birth were shown in the IVF group (6.67% vs 17.67%; 0 = 0.203). This result disagrees with a recent meta-analysis concluding that the risk of PTB in singleton pregnancies resulting from IVF/ICSI is significantly greater than that in spontaneously conceived singletons^10^. Given that the aim of our study wasn’t to show if PTB is more likely in IVF pregnancies, the design and sample size could explain why differences were not found.

Differences in richness and diversity in the microbiota between the two cohorts were reported. Although alpha diversity did not reach a statistical significance, we observed a trend through higher diversity in the IVF group was shown. As for, beta diversity difference in bacterial community composition between IVF and spontaneous cohorts was revealed. Increased richness and diversity of the vaginal microbiota and spontaneous preterm birth were reported^15,16^. As well, studies using sequencing approaches to detect microbes in fetal membranes, placenta and amniotic fluid of women with PTB have resulted in the detection of a wider diversity of microbial species^17^. Our result, may indicate that a diverse vaginal microbiome is more prevalent in IVF pregnant group at risk of PTB suggesting that the physiological state that leads to PTB might also create an environment that supports a richer/more diverse microbiota^15^.

In addition to differences in richness and diversity, differences in the microbiota between both cohorts regarding bacterial abundance and prevalence were also identified. The abundance of 4 taxa (*Gardnerella, Neisseria*, *Prevotella*, and *Staphylococcus*) was found to be over represented in vaginal microbiome at 12 weeks from patients from IVF pregnancies. An interesting finding was that all of the phylotypes that increased in abundance in IVF cohort were related to bacterial vaginosis. A compelling body of evidence supports a causal association between intra-amniotic infection and spontaneous preterm delivery^18^. The organisms found in the amniotic cavity are often similar taxonomically to those found in the lower genital tract of pregnant women as demonstrated by using both cultivation and molecular techniques^19^. Bacterial vaginosis, is a risk factor for spontaneous abortion, spontaneous preterm delivery, intra-amniotic infection, puerperal endometritis and adverse perinatal outcome^20^. Therefore, considering that an adverse pregnancy outcome may be caused by the ascension of pathogenic microbes, our results suggest that the microbiome composition could be the cause of higher risk of adverse pregnancy outcomes in IVF patients. Then, vaginal microbiome early in pregnancy may be most useful in the prediction of adverse outcomes.

Once we found differences in genus abundance composition between both cohorts, our last goal was to identify of microbial signatures that are predictive of IVF pregnancy at genus level, being an essential step toward the translation of vaginal microbiome research to obstetrical clinical practice. We used selbal, a greedy stepwise algorithm for the identification of microbial signatures consisting of two groups of taxa whose relative abundances, or balance, are predictive of the type of pregnancy. Working with balances and, in general, with log-contrast functions preserves the scale-invariant principle for compositional data analysis^21^. The microbial signature in the IVF group had lower balance scores than spontaneous group, which means that there are lower relative abundances of taxa *Acinetobacter* and *Enterococcus* than *Staphylococcus* and *Prevotella*. The genus *Enterococcus* is the most controversial group of lactic acid bacteria. The production of bacteriocins by enterococci is well documented^22^. Moreover, enterococci are nowadays used as probiotics^23^. *Acinetobacter* bacteria are ubiquitous found as natural inhabitants of human skin, gut and secretions and are considered to be normally found on many foods. Many acinetobacters acidify media, via an aldose dehydrogenase^24^. Once again the abundance of bacterial related of vaginosis and the shortage of protective ones, by acid lactic production, is a microbiome signature of IVF cohort patients suggesting that the microbiome composition could be the cause of higher risk of adverse pregnancy outcomes in this group of patients.

Regarding *Lactobacillus* composition, in the present study, we observed that the relative abundance of *L. iners* was higher in IVF samples, while the relative abundance of *L. gasseri* was higher in spontaneous samples. In other cohorts studying the role of microbiome in birth outcomes similar abundance profile of *L. gasseri* and *L. iners* have been reported in term and preterm samples^25^.

In a high-risk pregnant cohort, a strongly association among *L. iners* with short cervix and preterm birth was reported^26^. This finding has been confirmed by a recent study, suggesting that *L. iners* may play a role in the mechanisms of cervical shortening and modifying during pregnancy^27^. *L. iners* is the Lactobacilli specie with the lowest ability to neutralize infections from pathogens because *L. iners* produces d-lactate instead of l-lactate, low amounts of antimicrobial peptides, and has reduced ability to bind epithelial cells^28^. For these reasons, *L. iners* has reduced ability to prevent from bacterial vaginosis due to the enrichment of *Gardnerella* and other bacteria. Consequently *L. iners* has been suggested as a marker of microbial imbalance leading to bacterial vaginosis^29^. Moreover, it was reported that *L. iners* increases ectocervical and endocervical permeability, suggesting that this bacterial specie is less active in modulating inflammatory processes that could have negative consequences on cervical length during pregnancy^26^.

As for *L. gasseri,* it is significantly higher in women who delivered term babies compared to those who delivered preterm^30^. The presence of secretory transcriptional regulator and several ribosomally synthesized antimicrobial peptides reported by genomic analysis correlated with *L. gasseri* anti-inflammatory condition in the vagina. These findings indicate protective role of *L. gasseri* in reducing the risk of PTB. Also, in order to maintain vaginal homeostasis *L. gasseri* has a high potential as probiotics since they may confer colonization resistance against pathogens by direct inhibition through the production of antimicrobial toxins, as bacteriocins^31^. Due to our data, we cannot conclude that *L. iners* abundance is related to the IVF technique itself or by the patient’s infertility but, considering the previous investigations and the results of our study, we can assume that the presence of *L. iners* may accelerate some mechanisms potentially associated with preterm delivery, suggesting that the infertility patient PTB high risk of infertile patients might be associated to their vaginal microbiome composition. Similarly, *L. gasseri* carriage which is negatively associated with PTB has a protective role.

In our study, however, we did not identify a signature microbiota composition (CST) associated with pregnancy cohorts. Two reason could explain that the CST differences between groups could not be found although the abundance of *L. iners* and *L. gasseri* was different. Lower power due to lower sample size of women may have resulted in failure to observe differences between IVF and spontaneous pregnancy group. Moreover, differences in rare taxa that would differentiate both groups could be masked in CST assignments because they are defined by the dominance of a single species, and indeed, genus analysis revealed that the vaginal microbiota of women from the IVF group was richer and more diverse than those of women from the spontaneous pregnancy group.

The major strength of our study is that to the best of our knowledge, this is the first study that compares the vaginal microbiome of pregnant women at 12 weeks of gestation who conceived spontaneously or by means of through IVF. One of the main limitations of our study is that baseline samples prior to IVF or pregnancy were not collected. For future studies, it would be recommended to consider a baseline microbiome and to increase the cohort size in order to validate our findings. Our report focuses on relative abundance of different taxa, however, bacterial load quantification has not been determined. Although a 16S rRNA analysis is a powerful tool to characterize the composition of a microbial community, this approach provides limited information about the function and role of the vaginal microbial community. The use of a metagenomic approach would add considerable information. Finally, the study was not randomized and all patients enrolled in the study are Caucasian, thus limiting the conclusions to other populations.

Taken together, our results suggest that the microbiome vaginal signature at 12 weeks of gestation between women who conceived spontaneously or through IVF is different. The IVF cohort showed a signature of PTB including several taxa that have previously been implicated in adverse pregnancy outcomes. Thus, our findings contribute to understanding a possible reason why IVF pregnancy is at risk for PTB suggesting vaginal microbiome be the reason to the relation between IVF pregnancies and risk for PTB.

## Methods

### Ethics approval

The protocol was submitted and approved by the local Ethical Committee Hopsital Universitario de San Juan de Alicante (20/002 Tut). All research was performed in accordance with relevant guidelines and follows the Spanish Biomedical Law and Helsinki declaration. Also all participants received information concerning their participation in the study and provided written informed consent.

### Study design and subjects

Between January 2020 and June 2021 an observational, prospective and multicentric study was undertaken in two public hospitals and a private fertility clinic in Spain (Hospital Universitario San Juan de Alicante, Hospital General Universitario de Alicante and Instituto Bernabeu). A total of 64 Caucasian women were enrolled in the study. Two cohorts of women were selected: the first cohort of spontaneously pregnant and the second of women pregnant after an IVF treatment. The IVF group included patients which performed a controlled ovarian stimulation, oocyte retrieval, IVF or ICSI (intracytoplasmic sperm injection) fertilization and fresh embryo transfer. Exclusion criteria were: twin pregnancies, uterine and fallopian tubes malformations, suspicion of fetal malformation and/or chromosomopathy, vanishing twin, vaginal infections (vaginal discharge, itching, burning, pain, and a strong odor), early pregnancy bleeding and recent use of antibiotics. For each woman enrolled, vaginal swabs were collected at 12 weeks of gestation, in order to analyse the microbiome composition. In addition, clinical information of the participants during pregnancy and perinatal outcomes were collected. All participants were Caucasian.

### Sample collection, bacterial DNA extraction, 16S ribosomal RNA gene amplicon preparation, and illumina MiSeq sequencing

Vaginal swab collection was performed during a sterile speculum exam in the fornix posterior. The swabs used were dry and sterile (DELTALAB, Barcelona, Spain). The swabs were stored at room temperature using a DNA preservation medium (DNA/RNA Shield, ZYMO Research, Irvine, CA, USA) until they were processed.

Bacterial genomic DNA was extracted from the thawed vaginal samples by using the MagMAX™ CORE Nucleic Acid Purification Kit (Thermo Fisher Scientific, Madrid, Spain) and the automated extractor King-Fisher DUO Prime (Thermo Fisher Scientific, Madrid, Spain), following the manufacturer’s protocol. Quality control was carried out by gel electrophoresis and measuring ng/uL of DNA by using Qubit 4 Fluorometer (Thermo Fisher Scientific, Madrid, Spain) and the related Qubit dsDNA HS (High Sensitivity) assay kit.

The library of 16S rRNA gene amplicons was prepared through amplification of the V3-V4 hypervariable region by using specific-barcoded primers with overhanging adapters. The standard protocol was followed according to the 16 metagenomic sequencing library preparation guide from Illumina (Part # 15044223 Rev. B; https://support.illumina.com/). Participant samples were sequenced as were 10 no-template controls (that contained all assay components except for DNA to verify lack of contamination across reagents and samples). No sequences were obtained from no template samples, then contamination was ruled out. Pooled V3–V4 amplicon libraries were sequenced using the Illumina MiSeq platform.

### Sequencing analysis

Raw sequence data were analyzed using the QIIME 2 (v.2020.2.0) bioinformatics pipeline^32^. The 300-bp paired-end reads obtained from Illumina MiSeq platform for each sample were demultiplexed to attribute sequence reads to the appropriate samples and joined using vsearch. The sequence reads were then denoised and dereplicated into amplicon sequence variants (ASVs) using the deblur tool that also filtered out chimeras. Each sequence read was trimmed using a length of 450 bp. A total of 19,861,731 sequences of the 16S rRNA gene were generated from the 64 swab samples for a mean frequency of 310,340 sequences per sample. A feature table, which is equivalent to the OTU table generated by QIIME, was generated for all samples with a mean frequency per feature 5705. The feature table was then used to perform taxonomic classification, alpha and beta diversity analysis, and differential abundance measurements in different experimental groups. Taxonomy was assigned to each ASV using SILVA (version 138) database and a fitted classifier classify-sklearn method. Species-level assignments were based on the BLASTn software (https://blast.ncbi.nlm.nih.gov/)^33^. The highest percentage of identity and expectation value was considered to select significant BLAST hits.

### Statistical analysis

The primary outcome was the comparison of the vaginal microbiota between pregnant women who had conceived spontaneously or through IVF.

To test for differences in clinical variables we used the RStudio (v.1.4; R Core Team, 2021). We summarized sociodemographic and clinical characteristics of women according to her pregnancy (spontaneous or IVF). Chi-square was used for qualitative variables, and nonparametric test (Mann–Whitney U test) for quantitative variables, considering a p < 0.05 as statistical significance.

Sequence data analyses were performed in RStudio (v.1.4; R Core Team, 2021). Alpha diversity analysis, abundance and plot bars were performed by using phyloseq package v.1.34^34^. Principal coordinate analysis (PCoA) ordination (beta diversity) based on the unweighted unifrac distances and permutational multivariate analysis of variance (PERMANOVA) was calculated using QIIME2. Differential abundance for microbiome data was performed using DESeq2 package v.1.30^35^. Other packages used were metagenomeSeq package v.1.32^36^ and microbiomeSeq package v.0.1^37^. Selbal package^21^ was used for selection of balances in microbiome profile for different cohort of pregnant women.

According to the genus difference abundance between both cohorts, we moved forward the identification of a genus microbial signature related to each pregnancy group. The identification of microbial signatures involves both modelling and variable selection: modelling the response variable and identifying the smallest number of genus with the highest prediction or classification accuracy. We used selbal method that performs multiple regressions a number of times, each time adding a new genus to the model, the raw variables in selbal are added as part of what is called a “balance” in the compositional data analysis. This microbial signature is defined by two groups of genus whose relative abundances, or balance, are associated with cohort. Thus, the goal of selbal analysis was to identify a microbial signature, that is, groups of microbial genus for pregnancies that are able to discriminate between IVF and spontaneous pregnancies.

A community state type (CST) was assigned to each sample using hierarchical clustering with the NMDS bray Curtis. CST I is predominated by *L. crispatus*, CST II by *L. gasseri,* CST III by *L. iners,* CST IV is defined as lacking Lactobacillus predominance while CST V is predominated by *L. jensenii* as previously described^1^*.* Analysis of covariance (ANCOVA) was applied to compare IVF or spontaneous pregnancy among CSTs.

## Supplementary Information

Supplementary Figure S1.

Supplementary Figure S2.

Supplementary Table S1.
