# Supplementary figures and images for: Identification of vaginal microbiome associated with IVF pregnancy
Source: Sci Rep. 2022 Apr 26;12:6807. doi: 10.1038/s41598-022-10933-2 (PMC9042930; doi:10.1038/s41598-022-10933-2)

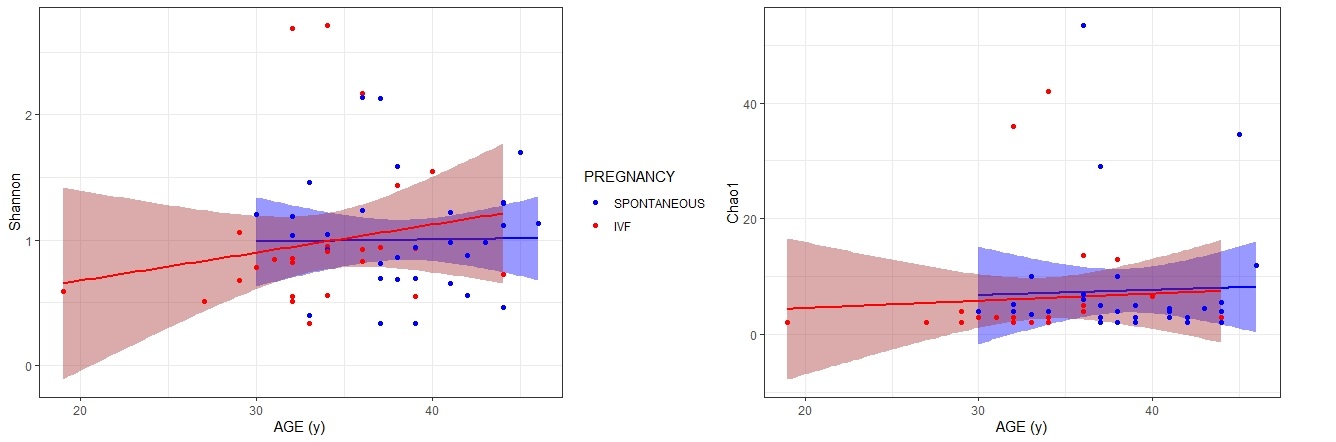

Supplement: Supplementary file 1 — Supplementary Figure S1. [file 41598_2022_10933_MOESM1_ESM.jpg]

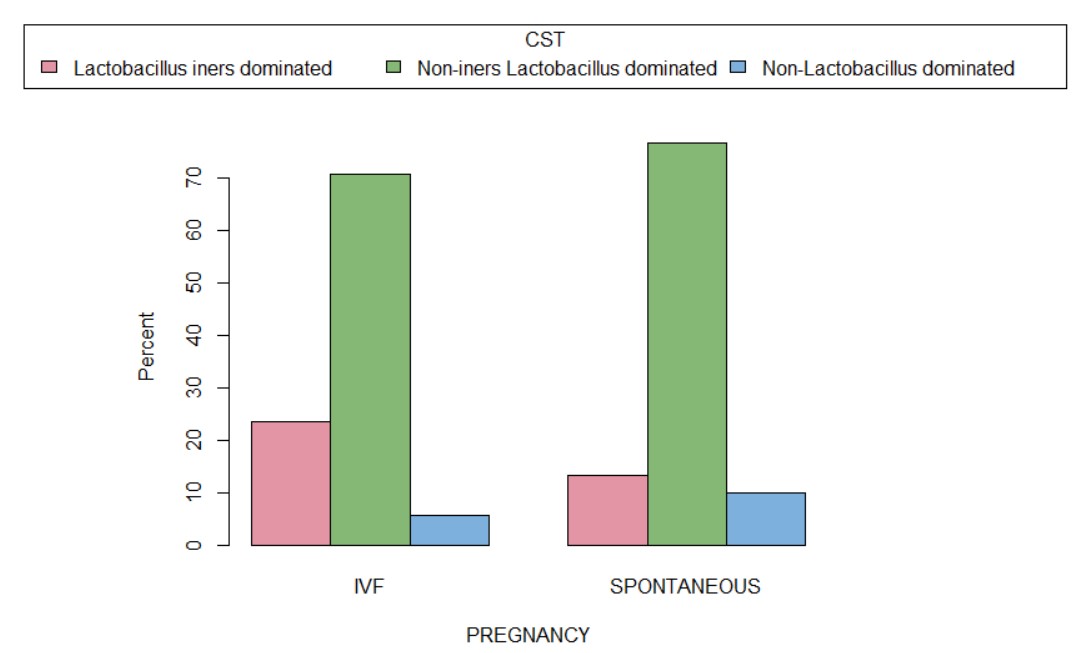

Supplement: Supplementary file 2 — Supplementary Figure S2. [file 41598_2022_10933_MOESM2_ESM.jpg]
