# Supplementary material for: Identification of vaginal microbiome associated with IVF pregnancy
Source: Sci Rep. 2022 Apr 26;12:6807. doi: 10.1038/s41598-022-10933-2 (PMC9042930; doi:10.1038/s41598-022-10933-2)
Supplement: Supplementary file 3 — Supplementary Table S1. [file 41598_2022_10933_MOESM3_ESM.docx]

**Table S1**. Distribution of CSTs in all recruited women, according to pregnancy

|  | All Women | Spontaneous pregnancy | IVF pregnancy | p-Value (Fisher’s Exact Test) |
| --- | --- | --- | --- | --- |
| N | 64 | 30 | 34 |  |
| CST I (%) | 48.4 | 46.7 | 50.0 | 0.650 |
| CST II (%) | 20.3 | 26.7 | 14.7 |  |
| CST III (%) | 18.8 | 13.3 | 23.5 |  |
| CST IV (%) | 7.8 | 10.0 | 5.9 |  |
| CST V (%) | 4.7 | 3.3 | 5.9 |  |
